# Oral health status among visually impaired schoolchildren in Northeast China

**DOI:** 10.1186/s12903-019-0752-2

**Published:** 2019-04-27

**Authors:** Lu Liu, Ying Zhang, Wei Wu, Mu He, Zhenfu Lu, Kaiqiang Zhang, Jian Li, Shuang Lei, Shibo Guo, Yuyu Zhang

**Affiliations:** 1 Department of Preventive Dentistry, School of Stomatology, China Medical University, Shenyang, 110002 China; 2 Department of Preventive Dentistry, Shanghai Stomatological Hospital, Fudan University, Shanghai, 200001 China; 3 Department of Epidemiology, School of Public Health, China Medical University, Shenyang, 110001 China; 4 Department of Pediatrics, Hangzhou Stomatological Hospital, Hangzhou, 310002 China; 5 XinXing Dental Clinic, HuangGu District, Shenyang, 110000 China

**Keywords:** Visual impairment, Schoolchildren, Oral health, Factors, China

## Abstract

**Background:**

Visual impairment is an important disability affecting a substantial proportion of people globally. The aim of this study was to assess the oral health status of visually impaired schoolchildren in northeast China, and to investigate the influencing factors.

**Methods:**

The study was performed in 2015, according to the criteria and methods used in the Third National Oral Health Epidemiological Survey in China. One hundred and three visually impaired schoolchildren from the only special school for the blind in northeast China were included in the study. Oral examinations were performed to assess the caries of deciduous and permanent teeth, periodontal disease, malocclusion. A questionnaire-based survey was conducted to investigate oral health-related behaviors, knowledge and attitude about oral care.

**Results:**

The overall prevalence of caries was 78.64%, and mean number of caries was 2.43 ± 2.75. The prevalence of caries in deciduous and permanent teeth was 65.22 and 71.84%, respectively. The rates of gingival bleeding and dental calculus were 44.66 and 67.96%, respectively. Malocclusion was observed in 49.51% of the children with visual impairment. The prevalence of caries was significantly higher in girls than boys (*P* < 0.05). The logistic regression analysis identified the knowledge level of parents and the toothache experience as risk factors for oral health, while the daily use of fluoride toothpaste could reduce the caries incidence.

**Conclusions:**

This group of visually impaired schoolchildren exhibited a high prevalence of dental caries, poor periodontal health, and severe malocclusion. Oral health status is relatively poor among visually impaired schoolchildren in northeast China. Factors that significantly affected the prevalence of dental caries included education level of the mother, experience of toothache, and use of fluoride toothpaste.

## Background

Visual impairment is an important disability affecting a substantial proportion of people globally. It is estimated that over 1.4 million children worldwide are living with visual impairment; 75% of this population live in the poorest regions of Asia and Africa [1]. The prevalence of visual impairment in children in some low-income countries and regions reaches 0.15% [1, 2]. If visual perception is damaged by disease or drugs, cognition is also affected, which can impact oral health [2].

Oral health is an important component of systemic health. Oral disease may be difficult to diagnose in disabled children, and adherence to treatment may be poor [1, 3–5]. In addition, the lack of hand-eye coordination, inadequate parental supervision, and a lack of input from peers can reduce attention to oral health [1, 6].

However, few studies have investigated oral health in visually impaired children, most of whom live in the poorest regions of Asia and Africa [1]. Recent studies have shown that the prevalence of caries in visually impaired children may be as high as 40–81.9% [1, 7–10], and the Oral Hygiene Index-Simplified (OHI-S) index is as low as 1.5–2.72 [1, 2, 11]. For instance, a study found that the Oral Hygiene Index-Simplified (OHI-S) index in 50 visually impaired children was only 2.72 [12]. One reason for the high prevalence of caries among visually impaired children may be the use of milk and cookies as a reward for good behavior [1, 7, 10]. Limited capacity for plaque removal and insufficient surveillance could also increase the prevalence of caries [8]. Therefore, we must identify the key factors influencing the oral health status of visually impaired children.

China is the most populous developing country, where 0.14 million schoolchildren were living with visual impairment on April 1, 2006 [11]; 79.07% are educated in regular or special schools [11]. A study that investigated the oral health and related behaviors of visually impaired schoolchildren in South China showed that the prevalence of caries in permanent teeth was 54.7% among those aged 12 years [13]. In the same group, mean number of caries was 1.53, which was higher than the results reported by the Third National Oral Health Epidemiological Survey in China [14]. Another study conducted in Central China showed that the periodontal health status of visually impaired children was relatively poor; the rates of gingival bleeding and dental calculus were higher than in age-matched normal children in the same region [15].

These issues remain to be investigated among visually impaired schoolchildren in northeast China. Northeast China represents a juncture between the Circum-Bohai Sea Economic Zone and the Northeast Economic Zone, which is a center of economy, politics, and culture. However, the community oral health services in China remain insufficient, especially in the Northeast, where development lags behind that of coastal regions. Therefore, it is extremely urgent to establish medical and oral healthcare services system. Furthermore, no comprehensive health security system for disabled persons has been established in northeast China. Visually impaired children have only limited access to oral healthcare services.

The present study aimed to investigate the oral health status, as well as the associated factors, of visually impaired schoolchildren enrolled at the only school for the blind in northeast China. In this study, cluster sampling was used to recruit participants from the only special school for the blind in northeast China. Oral examinations and questionnaire surveys were conducted; data related to oral health, oral behaviors and habits were analyzed to identify factors affecting oral health. This study will provide evidence to help optimize the distribution of oral health sources and garner attention to oral health in disabled children.

## Methods

### Subjects

All 119 visually impaired children from the only school for the blind in northeast China were recruited, among which 16 were excluded because their guardians were either unavailable or did not agree to participate in the study. Ultimately, 103 of the schoolchildren were investigated including 70 males and 33 females, with the mean age of 15.93 ± 4.28 years (range, 6–20 years). Visual impairment was classified to one of four categories (grade 1, 2, 3, and 4. Grade 1 and 2 were considered blind, and grade 3 and 4 were considered low vision), according to pre-defined criteria [16]. Oral examinations and questionnaires were administered after written informed consent was obtained from the parents and teachers. This study was approved by the Ethics Committee of the School of Stomatology, China Medical University.

### Data collection

According to the criteria and methods used in the Third National Oral Health Epidemiological Survey in China [14], oral examinations were conducted in May 2015. In brief, a senior oral physician was asked to assess the patient’s oral health status, with the help of an artificial light source. The examination proceeded from the first to the fourth quadrants using a plane dental mirror and CPI periodontal probe (force≈20 *g*). The oral health examination in each visually impaired child included DMFT for assessment of dental caries, gingival bleeding and dental calculus for evaluating periodontal disease, occlusion indices for recording malocclusion.

A questionnaire was used to collect data on general characteristics (registered residence, gender, and ethnicity), oral health-related behaviors, use of fluoride-containing toothpaste, dietary habits, healthcare-seeking behaviors, and knowledge and attitudes about oral healthcare.

### The calibration

All investigators were uniformly trained before the investigation; the consistency test yielded a kappa value > 0.90. After 15% of visually impaired children were re-examined, comparison of the results yielded a kappa value = 0.86. The questionnaire survey was administered in the context of a face-to-face interview by uniformly trained investigators, who also recorded the answers and then collected the questionnaires.

### Statistical analysis

Data were entered by two investigators independently with Epidata 3.0 software (The Epidata Association, Odense, Denmark). The data were analyzed with SPSS 13.0 (SPSS Inc., Chicago, IL) after logic verification. A t-test was used for comparison of continuous variables. Qualitative data were compared with univariate analysis (chi-square test) to identify possible risk factors for dental caries. Multivariate binary logistic regression analysis was used to identify variables significant for various risk factors. Differences with *P* < 0.05 were considered statistically significant.

## Results

### General characteristics

Among 119 children who attended a special school for the visually impaired, 16 were excluded because their guardians were either unavailable or did not agree to participate in the study. Ultimately, 103 of the schoolchildren were included. The study participants included 70 males and 33 females. Sixty-six of the children had grade-1 or − 2 visual impairment (referred to as blind), and the other 37 had grade-3 or − 4 visual impairment (low vision). The mean age of the children was 15.93 ± 4.28 years (range, 6–20 years).

### Caries

The overall mean number of caries was 2.43 ± 2.75; the prevalence of caries was 78.64%. The mean number of caries was higher in females than in males (*P* < 0.05) (Table 1). The prevalence of caries-free children was 21.36%.

**Table 1:** Overall status of dental caries in 103 schoolchildren

Variables		n^a^ (%)	Mean number of caries	Caries prevalence rate
Gender	M	70 (68%)	2.16 ± 2.07*	77.14%
	F	33 (32%)	3.12 ± 3.82	81.82%
Age (years)	6–12	25 (24%)	3.2 ± 2.87	84%
	13–20	78 (76%)	2.23 ± 2.76	76.92%
Status of visual impairment	Low vision	37 (36%)	1.68 ± 1.84	72.97%
	Blind	66 (64%)	2.85 ± 3.06	81.82%
Total (%)		103 (100%)	2.43 ± 2.75	78.64%

*P < 0.05, comparing with the females. ^a^number of children

The prevalence of caries in deciduous teeth was 65.22%, and the mean number of dental caries was 2.17 ± 2.41. The prevalence of caries in permanent teeth was 71.84%; the mean number of dental caries was 1.95 ± 2.50. For caries in deciduous teeth, 96% of teeth were missing; only 4% of the caries were filled. For caries in permanent teeth, 3% of teeth were missing, and only 13% of caries had been filled. In addition, only 11.6% of the mixed dentition with caries underwent filling (Table 2).

**Table 2:** Numbers, percentages, and statistical data of DMF index

	Dental caries			Missing due to caries			Filling due to caries		
	n^a^	%	Mean ± SD	n^a^	%	Mean ± SD	n^a^	%	Mean ± SD
Deciduous teeth	47	96%	2.04 ± 2.36	0	0%	0	2	4%	0.13 ± 0.46
Permanent teeth	168	84%	1.63 ± 1.97	6	3%	0.06 ± 0.34	27	13%	0.26 ± 0.84
Mixed dentition	215	86%	2.09 ± 2.32	6	2.4%	0.06 ± 0.34	29	11.6%	0.28 ± 0.86

Caries of the first permanent molar and pit and fissure sealant

^a^number of teeth

All the children included had at least one first permanent molar eruption. The overall prevalence of caries in the first permanent molar was 47.57%. The mean number of decayed first permanent molars was 0.82 ± 0.99 per child. The mean number of decayed first permanent molars in the low-vision group was significantly lower than in the blind group (*P* < 0.05). The prevalence of pit and fissure sealant use was only 0.97% (Table 3).

**Table 3:** Caries of the first permanent molar and use of pit and fissure sealant

Variables	Gender		Age (years)		Status of visual impairment		Total
	M	F	6–12	13–20	Low vision	Blind	
Numbers	70	33	25	78	37	66	103
Number of children with the first permanent molar	280	132	100	312	148	264	412
Number of children with caries of the first permanent molar	33	16	13	36	16	33	49
Prevalence of caries of the first permanent molar (%)	47.14	48.48	52.00	46.15	43.24	50.00	47.57
Number of caries of the first permanent molar	54	29	23	61	22	62	84
Mean number of caries of the first permanent molar	0.79 ± 0.98	0.88 ± 1.05	0.92 ± 1.04	0.78 ± 0.99	0.59 ± 0.76*	0.94 ± 1.09	0.82 ± 0.99
Number of children with pit and fissure sealant	1	0	0	1	0	1	1

^*^P < 0.05, comparing with the blind group

### Gingival bleeding and dental calculus

Among the study participants, 247 teeth showed gingival bleeding; the mean number of teeth with gingival bleeding was 2.40 per child. The prevalence of gingival bleeding was 44.66%. A total of 410 teeth showed dental calculus, and the mean number of teeth with dental calculus was 3.98 per child. The mean prevalence of dental calculus was 67.96%. The prevalence of dental calculus differed significantly between age groups (*P* < 0.05) (Table 4).

**Table 4:** Gingival bleeding and dental calculus of the 103 children

Variables		N	Number of children with gingival bleeding	%	Number of children with dental calculus	%
Gender	M	70	33	47.14	47	67.14
	F	33	13	39.39	23	69.7
Age (years)	6–12	25	9	36.00	13	52.00*
	13-20	78	37	47.44	57	73.08
Status of visual impairment	Low vision	37	16	43.24	30	81.08
	Blind	66	30	45.45	40	60.61
Total		103	46	44.66	70	67.96

^*^P < 0.05, compared with the 13–20-year-old group

### Malocclusion

The prevalence of malocclusion among the study population was 49.51%. The prevalence of malocclusion was significantly higher in girls than boys (P < 0.05). The prevalence of anterior crossbite in the study population was 22.33%. The prevalence of dental crowding and deep overbite/overjet were 12.62 and 11.65%, respectively. The prevalence of others malocclusions (including open bite, posterior crossbite, cleft lip and palate, etc) was 2.91% (Table 5).

**Table 5:** Prevalence of malocclusion

Variables		N	Anterior crossbite (%)	Dental crowding (%)	Deep overbite/overjet (%)	Others malocclusions (%)	Total (%)
Gender	M	70	15 (21.43%)	7 (10%)	4 (5.71%)	1 (1.24%)	28 (40.00%)*
	F	33	8 (24.24%)	6 (18.18%)	8 (24.24%)	2 (6.06%)	23 (69.70%)
Age (years)	6–12	25	3 (12%)	5 (20%)	2 (8%)	1 (4%)	12 (48.00%)
	13–20	78	20 (25.64%)	8 (10.26%)	10 (12.82%)	2 (2.56%)	39 (50.00%)
Status of vision impairment	Low vision	37	11 (29.73%)	7 (18.92%)	0 (0%)	0 (0%)	18 (48.65%)
	Blind	66	12 (18.18%)	6 (9.09%)	12 (18.18%)	3 (4.55%)	33 (50.00%)
Total (%)		103	23 (22.33%)	13 (12.62%)	12 (11.65%)	3 (2.91%)	51 (49.51%)

^*^P < 0.05, compared with the female group

Others malocclusions, include open bite, posterior crossbite, cleft lip and palate, etc.

### Univariate analysis of factors affecting dental caries

In order to identify factors affecting dental caries, we analyzed data collected using the questionnaire (including age, gender, family economic status, knowledge of oral health, oral health-related habits and behaviors, and social background) with the univariate chi-square test. The results showed that only the education level of the mother (*χ*^2^ = 7.68, *P* = 0.005), use of fluoride toothpaste (*χ*^2^ = 4.75, *P* = 0.029), and toothache within the past year (*χ*^2^ = 8.62, *P* = 0.003) significantly affected the status of dental caries in visually impaired schoolchildren (Table 6).

**Table 6:** Univariate analysis of potentially significant factors

Factors	Group	n	Number of children with caries	*χ*2	*P*
Mother’s education level < middle school	Y	79	67		
	N	24	14	7.68	0.005
Toothache within the past year	Y	52	47		
	N	51	34	8.62	0.003
Using fluoride-containing toothpaste	Y	69	50		
	N	34	31	4.75	0.029

### Multivariate logistic regression analysis of dental caries

Caries status was included as the dependent factor, and the factors suggested by the univariate chi-square test, including the education level of the mother, use of fluoride toothpaste, and toothache within the past year, were included as independent factors (together with age and gender) for the multivariate logistic regression analysis. For children without dental caries, a score of “0” was assigned; for children with dental caries, a score of “1” was assigned. The results showed that the education level of the mother (OR = 3.827, 95% CI = 1.165–12.570), use of fluoride toothpaste (OR = 0.196, 95% CI = 0.039–0.986), and experience of toothache within the past year (OR = 4.781, 95% CI = 1.360–16.805) significantly affected caries status among the study population (Table 7). Among these factors, lower education level of the mother and experience of toothache within the past year were risk factors, while use of fluoride toothpaste protected against dental caries.

**Table 7:** Multivariate logistic regression analysis of prevalence of dental caries

Factors	β	S.E.	Wald	OR	95%CI
Mother’s education level	1.342	0.607	4.893	3.827	1.165–12.570
Experience of toothache within the past year	1.565	0.641	5.950	4.781	1.360–16.805
Using fluoride toothpaste	−1.628	0.823	3.908	0.196	0.039–0.986

## Discussion

As visually impaired schoolchildren in China do not yet have access to healthcare, their oral health status remains a pressing issue [13, 15]. In this study, cluster sampling was performed to recruit participants after the school permitted participation in our investigation. Our findings showed that the prevalence of dental caries (78.64%) and mean number of caries (2.43 ± 2.75) were high as comparing with the results reported by previous studies. The prevalence of dental caries and mean number of caries among students aged 6–12 years (84.00% and 3.20 ± 2.87, respectively) were substantially higher than the results reported for age-matched controls by the third national oral health epidemiological survey in China [14] and other studies [2, 10, 13, 17, 18], but in agreement with results reported by Hou et al. in Guangxi province [19]. We also found that the prevalence of caries was significantly higher in females than in males (*P* < 0.05), as reported previously [20]. We speculated that this gender difference could be associated with earlier tooth eruption in girls than in boys, which could result in protracted exposure to adverse factors and, thus, an increase risk of caries.

The Third National Oral Health Epidemiological Survey in China [14] has shown that the rates of gingival bleeding and dental calculus are inversely associated with the frequency of tooth brushing in normal children; children who brush their teeth at least twice per day have the lowest risk of gingival bleeding and dental calculus. In the present study, we found a higher rate of gingival bleeding (44.66%), mean number of teeth with gingival bleeding (2.40 per child), rate of dental calculus (67.96%), and mean number of teeth with dental calculus (3.98 per child) in visually impaired children compared with children in the general population, as reported previously [13, 17–19]. The high rates of gingival bleeding and dental calculus in visually impaired children were associated with a poor understanding of how to brush the teeth appropriately, which could result in insufficient clearing of the teeth [20]. The rate of dental calculus was also significantly higher in older compared with younger children, suggesting that the amount of dental calculus accumulates with age. These findings show that visually impaired children generally have poor oral health, with an especially high prevalence of dental calculus, suggesting that early oral health education (e.g., how to appropriately brush the teeth) in visually impaired children could help reduce the risk of oral disease and improve oral health status.

Previous studies have reported that the prevalence of malocclusion in children is about 37.61–72.92% [21, 22]. The present study showed that the rate of malocclusion in visually impaired children was 49.51%. The rate of anterior crossbite reached 22.33%, and the rates of dental crowding and deep overbite/overjet were 12.62 and 11.65%, respectively (similar to levels observed in children in the general Chinese population) [14]. A previous study has shown that the prevalence of malocclusion is positively associated with oral health behaviors [21], and poor oral habits are a major cause of malocclusion [23]. The frequency, duration, and intensity of poor oral health habits may significantly affect the severity of malocclusion [23]. When habits (such as mouth-breathing) that can lead to malocclusion develop in visually impaired children, they should be corrected in a timely fashion to prevent the development and progression of malocclusion and thus improve the child’s oral health.

The results of the multivariate logistic regression analysis also showed that low level of maternal education is a risk factor for dental caries. Previous studies have shown that the education level of the parents is an important factor influencing the oral health behaviors of children [22]. For instance, the attitudes of the children regarding eating desserts and self- oral examination differ significantly among children, depending on the parents’ level of education. Children of parents with higher education levels have more opportunities to acquire knowledge about oral healthcare and related health education, which can enhance active participation in early oral healthcare. Parents with relatively lower education levels may ignore the oral health of their children and fail to intervene appropriately when an issue arises [24].

The present study also showed that the experience of toothache is also risk factor for dental caries in visually impaired children, as reported previously [13]. Visual impairment may impede tooth-brushing or flossing, which can increase the risk for dental caries [2]. Asymptomatic oral disorders may also be ignored by visually impaired children. When symptoms develop, visually impaired children may not receive the care they need because they lack medical insurance. The rate at which visually impaired children seek medical attention for oral issues is low (only 22.33%) [14, 15].

Multivariate logistic regression analysis showed that the use of fluoride toothpaste protected against the development of dental caries in visually impaired children. The American Dental Association has recognized that using the appropriate amount of fluoride can reduce the incidence of dental caries and decelerate the progression of dental caries [25, 26]. Previous studies have used fluoride exposure as an important index in assessing the risk for dental caries [27, 28]. These findings suggest that oral health education for visually impaired schoolchildren should include how to appropriately use oral health products, including fluoride toothpaste, to improve oral health status.

Certain limitations of this study should be acknowledged. The findings of this study could be biased by the relatively small sample size. However, although we applied to perform this study in several schools for disabled children, only this school permitted this study. Therefore, only only a small fraction of visually impaired children were included in this study. Visually impaired children who are home-schooled were not recruited for this study. Nonetheless, the findings of this study demonstrate poor oral health status among visually impaired schoolchildren in northeast China. Factors that significantly affect the development and progression of dental caries in visually impaired schoolchildren include the education level of the mother, toothache in the past year, and use of fluoride toothpaste. More studies with larger sample sizes are needed to further verify our findings.

## Conclusions

In summary, visually impaired schoolchildren have only limited access to oral healthcare. Cognitive deficits may limit such children from actively preventing dental caries. The findings of this study showed that the prevalence of dental caries, as well as the incidence of dental calculus and malocclusion, were higher among visually impaired schoolchildren as compared with children in the general population, highlighting the need to improve access to healthcare services among disabled children. In the further research, efforts should focus on improving oral health education for disabled children, educating the parents of disabled children about oral healthcare, and increasing the rate of topical fluoridation and other appropriate caries-preventing methods.
